# SARS-CoV-2 seroprevalence, seroconversion and neutralizing antibodies in a systemic lupus erythematosus cohort and comparison to controls

**DOI:** 10.1177/09612033211063793

**Published:** 2021-12-07

**Authors:** Hannah R Mathew, May Y Choi, Katherine Buhler, Xenia Gukova, Francesca S Cardwell, Heather Waldhauser, Ann E Clarke, Marvin J Fritzler

**Affiliations:** 1 Cumming School of Medicine, 2129 University of Calgary, Calgary, AB, Canada; 2 8430 University of Waterloo, Waterloo, ON, Canada

Sir,

At the outset of the SARS-CoV-2 pandemic, it was speculated that systemic lupus erythematosus (SLE) patients may be at significant risk of COVID-19 due to underlying immune dysregulation and immunosuppressive therapies (reviewed in^1^). It was unclear how these factors would alter B/T cell responses, risk of infection, and/or development of neutralizing antibodies.^2,3^ In this study, we examined the prevalence of SARS-CoV-2 antibodies using multiple assays, RT-PCR positivity, and neutralizing antibodies in 173 SLE patients (94.8% female, mean age 48.5 years, mean disease duration 11.7 years, 42.8% non-White race/ethnicity, 83.2% prescribed hydroxychloroquine, 28.9% corticosteroids, and 43.9% other immunomodulators) prior to vaccination compared to controls.

Pre-pandemic serum samples biobanked prior to 01/01/2020 and intra-pandemic samples collected from 03/15/2020–01/31/2021 were tested for SARS-CoV-2 antibodies using an ELISA measuring IgA and IgG anti-spike 1 (S1) protein (Euroimmun AG, Lübeck, Germany) and an assay detecting IgG antibodies to nucleocapsid (N), S1 receptor binding domain (RBD), and S1 (XMAP®: Luminex Corporation, Austin, TX) and conventional SLE autoantibodies (anti-Ro52, -SSA/Ro60, -SSB/La, -Sm, -U1RNP, -ribosomal P, and -dsDNA). One hundred pre-pandemic and 148 intra-pandemic sera (i.e., 248 unique individuals) from unselected ambulatory individuals undergoing autoantibody testing served as controls. RT-PCR tests were performed on the SLE cohort if clinically indicated and results retrospectively collected until 01/31/2021. Pre-pandemic and intra-pandemic SLE and control samples with antibodies to at least one SARS-CoV-2 antigen were tested for neutralizing antibodies using the Surrogate Virus Neutralization Test (GenScript Biotech Corporation, Piscataway, NJ, USA).^4^

None of the SLE patients had pre-pandemic SARS-CoV-2 antibodies versus 6% of controls (difference −6.0%, 95% CI: −10.7%, −1.4%; Table 1). Comparable proportions of SLE patients and controls had at least one intra-pandemic SARS-CoV-2 antibody (3.5% versus 4.7%, difference −1.2%, 95% CI: −5.6%, 3.2%). A sensitivity analysis age and sex-matching controls to SLE patients (2:1) yielded similar results. Intra-pandemic seroprevalence of IgG antibodies to the N protein in SLE patients was lower than in the general population (Calgary, AB Canada) over a similar observation interval^5^ (0.6% vs 2.9%, difference −2.3%, 95% CI: −3.6%, −1.0%). 7.5% (6/80) of SLE patients had a positive RT-PCR. None of the 173 SLE patients in the cohort, including the nine SLE patients with either intra-pandemic SARS-CoV-2 antibodies and/or a positive RT-PCR (Supplementary Table 1) were hospitalized for SARS-CoV-2 infection.

**Table 1.** SLE cohort patients and controls with pre- and/or intra-pandemic SARS-CoV-2 antibodies.^a^

			SLE, n (%) (*n* = 173)	Controls, n (%) (pre-pandemic, *n* = 100; intra-pandemic, *n* = 148)	Difference between cohort and controls, % (95% CI)
Pre-pandemic	ELISA	*Anti-S1 IgA* ^b^	0 (0.0)	3 (3.0)	−3.0 (−6.3, 0.3)
		*Anti-S1 IgG* ^c^	0 (0.0)	4 (4.0)	−4.0 (−7.8, 0.1)
	xMAP®	*Anti-N IgG* ^d^	0 (0.0)	2 (2.0)	−2 (−4.7, 0.7)
		*Anti-RBD IgG* ^d^	0 (0.0)	0 (0.0)	0. (0.0, 0)
		*Anti-S1 IgG* ^d^	0 (0.0)	0 (0.0)	0. (0.0, 0)
	Patients with at least one antibody^e^		0 (0.0)	6 (6.0)	**−6.0 (-10.7, -1.4)**
	Neutralizing antibodies^f^		0 (0.0^g^)	0 (0.0^g^)	NA
Intra-pandemic	ELISA	*Anti-S1 IgA* ^b^	1 (0.6)	3 (2.0)	−1.4 (−3.9, 1.1)
		*Anti-S1 IgG* ^c^	5 (2.9)	5 (3.4)	−0.5 (−4.3, 3.3)
	xMAP®	*Anti-N IgG* ^d^	1 (0.6)	5 (3.4)	−2.8 (−5.9, 0.3)
		*Anti-RBD IgG* ^d^	2 (1.2)	3 (2.0)	−0.8 (−3.6, 2.0)
		*Anti-S1 IgG* ^d^	0 (0.0)	1 (0.7)	**−0.7 (-11.1, -2.9)**
	Patients with at least one antibody^e^		6 (3.5)	7(4.7)	−1.2 (−5.6, 3.2)
	Neutralizing antibodies^f^		2 (33.3)	4 (57.1)	Not done^h^
	RT-PCR (+/−)		6 (7.5^i^)	NA	

Bolded differences indicate statistical significance.

ELISA: enzyme-linked immunosorbent assay; IgA: immunoglobulin A; IgG: immunoglobulin G; N: nucleocapsid; NA: not applicable; RBD: receptor binding domain; RT-PCR: reverse transcription-polymerase chain reaction; S1: spike 1 protein; SLE: systemic lupus erythematosus; XMAP: Luminex addressable laser bead imunoassay.

^a^None of the SLE patients received a COVID vaccination prior to intra-pandemic testing. None of the controls received a COVID vaccination prior to pre-pandemic sampling. We were unable to verify the vaccination status of the intra-pandemic control samples, but there was very limited vaccine available in Canada prior to 31 January 2021.

^b^Cut-off for positivity: 1.9 OD ratio.

^c^Cut-off for positivity: 0.8 OD ratio.

^d^Cut-off for positivity: 700 MFI.

^e^identified by either ELISA or xMAP® assays.

^f^Cut-off for positivity: 20%.

^g^Only patients positive for SARS-CoV-2 antibodies pre- and/or intra-pandemic were assessed for neutralizing antibodies (pre-pandemic, *n* = 6 controls, *n* = 0 SLE; intra-pandemic, *n* = 7 controls, *n* = 6 SLE).

^h^Statistical testing not done due to low *n*.

^i^80 patients had a RT-PCR performed.

Two of six SLE patients with at least one SARS-CoV-2 intra-pandemic antibody developed neutralizing antibodies (medication profiles in Supplementary Table 1); both had IgG antibodies to the RBD of SARS-CoV-2 (Supplementary Table 2). None of six controls with at least one pre-pandemic antibody to SARS-CoV-2 had neutralizing antibodies, whereas 4/7 controls with at least one intra-pandemic SARS-CoV-2 antibody had neutralizing antibodies, three of which had IgG antibodies to RBD. As shown in Supplementary Table 3, there was no statistical difference (chi-squared test) in frequency of any pre- or intra-pandemic SLE-related autoantibodies between SLE patients with and without SARS-CoV2 positivity. This and the absence of SARS-CoV2 antibodies in pre-pandemic sera suggests that molecular mimicry is an unlikely explanation for SARS-CoV-2 seropositivity.^6^

Like other reports,^3,7,8^ our SLE cohort had a lower rate of seropositivity pre-pandemic and a slightly lower to similar rate of seropositivity intra-pandemic compared to contemporaneous controls. In contrast, over a similar observation period, others reported that 4% (4/100) SLE patients had PCR-confirmed infection, but 36% showed SARS-CoV-2 antibodies of at least one isotype, particularly IgA and IgM.^2^ However, these antibodies were also detected in pre-pandemic samples and had low neutralizing activity. We also measured IgM antibodies but found them to be an unreliable indicator of SARS-CoV-2 exposure and IgA cut-offs needed to be increased according to local controls. Although there is emerging evidence of higher rates of SARS-CoV2 infections and increased odds of mortality in rheumatic disease patients,^9^ it is unclear which factors influence SARS-CoV-2 infection in SLE,^2,3,7,8.^ However, as no pre-pandemic SARS-CoV2- IgG antibodies were observed in our SLE cohort, this seems an unlikely explanation for protection against COVID-19. Current efforts are focusing on vaccine responses in SLE.^10,11^

## Supplemental Material

sj-pdf-1-lup-10.1177_09612033211063793 – Supplemental Material for SARS-CoV-2 seroprevalence, seroconversion and neutralizing antibodies in a systemic lupus erythematosus cohort and comparison to controls Click here for additional data file. Supplemental Material, sj-pdf-1-lup-10.1177_09612033211063793 for anSARS-CoV-2 seroprevalence, seroconversion and neutralizing antibodies in a systemic lupus erythematosus cohort and comparison to controls by Hannah R Mathew, May Y Choi, Katherine Buhler, Xenia Gukova, Francesca S Cardwell, Heather Waldhauser, Ann E Clarke, and Marvin J Fritzler in Lupus
