# Supplementary material for: SARS-CoV-2 seroprevalence, seroconversion and neutralizing antibodies in a systemic lupus erythematosus cohort and comparison to controls
Source: Lupus. 2021 Dec 7;30(14):2318–20. doi: 10.1177/09612033211063793 (PMC8761959; doi:10.1177/09612033211063793)
Supplement: sj-pdf-1-lup-10.1177_09612033211063793 – Supplemental Material for SARS-CoV-2 seroprevalence, seroconversion and neutralizing antibodies in a systemic lupus erythematosus cohort and comparison to controls [file sj-pdf-1-lup-10.1177_09612033211063793.pdf]

Supplemental Tables: SARS-CoV-2 Seroprevalence, Seroconversion and Neutralizing Antibodies in a Systemic Lupus Erythematosus Cohort and Comparison to Controls

Supplemental Table 1. Expanded SLE cohort patients with positive intra-pandemic SARS-CoV-2 antibodies and/or positive RT-PCR

ID	Month of PCR Test	Month of Serum collection	Age	Sex	Race/ Ethnicity	Medications	
						At Time of Pre-pandemic Serum Sample	At Time of Intra-pandemic Serum Sample
**SARS-CoV-2 Antibody + and RT-PCR+**							
**SLE-A**	**Oct '20**	**Oct '20**	**40-45**	**M**	**Asian**	**hydroxychloroquine**	**hydroxychloroquine**
**SLE-B**	**Dec '20**	**Jan '21**	**40-45**	**F**	**Asian**	**hydroxychloroquine, mycophenolate mofetil, corticosteroids**	**hydroxychloroquine, mycophenolate mofetil, belimumab**
**SLE-C**	**Apr '20**	**Oct '20**	**45-50**	**F**	**Asian**	**hydroxychloroquine, mycophenolate, corticosteroids**	**hydroxychloroquine, mycophenolate**
**SARS-CoV-2 Antibody + and RT- PCR Not Done**							
SLE-D	ND	Oct '20	60-65	F	White	none	none
SLE-E	ND	Oct '20	45-50	F	White	hydroxychloroquine, methotrexate	hydroxychloroquine, methotrexate
SLE-F	ND	Oct '20	70-75	F	White	hydroxychloroquine	hydroxychloroquine
**SARS-CoV-2 Antibody - and RT-PCR +**							
SLE-G	Dec '20	Nov '20	20-25	F	White	hydroxychloroquine, azathioprine	hydroxychloroquine, mycophenolate mofetil
SLE-H	Oct '20	Jun '20	50-55	F	Asian	mycophenolate mofetil, corticosteroids	methotrexate
SLE-I	Nov '20	Sep '20	45-50	F	White	hydroxychloroquine	hydroxychloroquine

Bolded patients were positive for neutralizing antibodies. Only those positive for SARS-CoV-2 antibodies were assessed for neutralizing antibodies.

Supplemental Table 2. Expanded SLE cohort patients and controls with pre- and/or intra-pandemic SARS-CoV-2 antibodies. Boldface and cell shading indicate positive results.

	**Pre-Pandemic**							**Intra-Pandemic**						
		**ELISA**		**xMAP®**					**ELISA**		**xMAP®**			
		**S1 IgA OD [1]**	**S1 IgG OD [2]**	**N IgG IU [3]**	**RBD IgG IU [4]**	**S1 IgG IU [5]**	**NABs % inhibition [6]**		**S1 IgA OD [1]**	**S1 IgG OD [2]**	**N IgG IU [3]**	**RBD IgG IU [4]**	**S1 IgG IU [5]**	**NAB % inhibition [6]**
**SLE Cohort**	SLE-A (Ro52+)	0.2	0.5	0.90	1.11	13.76	Negative	SLE-A (Ro52+)	0.2	**3.5**	1.02	1.11	13.76	Negative
	**SLE-B (Ro52+)**	0.4	0.2	3.07	3.35	22.95	Negative	**SLE-B (DNA+)**	**>10**	**2.1**	**94.26**	**1495.04**	231.91	**High Positive (96.2%)**
	**SLE-C (DNA+)**	0.1	0.3	15.56	1.95	22.95	Negative	**SLE-C (neg)**	0.2	0.2	*29.08*	**269.41**	32.14	**Positive (66.7%)**
	SLE-D (neg)	0.2	0.3	7.05	1.39	27.54	Negative	SLE-D (neg)	0.8	**2.7**	*8,98*	2.79	27.54	Negative
	SLE-E (not tested)	0.8	0.6	1.15	1.11	87.48	Negative	SLE-E (Ro52+, Ro60+, La+)	1.7	**2.5**	1.79	1.39	129.20	Negative
	SLE-F (neg)	0.2	0.1	4.23	1.39	18.35	Negative	SLE-F (neg)	0.2	**0.9**	2.18	1.11	18.35	Negative
	**Total Positive (n (%[7]))**	*0 (0.0)*	*0 (0.0)*	*0 (0.0)*	*0 (0.0)*	*0 (0.0)*	*0 (0.0)[8]*	**Total Positive (n (%[7]))**	*1 (0.6)*	*5 (2.9)*	*1 (0.6)*	*2 (1.2)*	*0 (0.0)*	*2 (33.3)[8]*

**Control Cohort**	**CON-A**	0.2	0.4	**102.00**	1.67	115.27	Negative	**CON-G**	0.9	**4.3**	**324.04**	**491.36**	236.61	**High Positive (94.6%)**
	**CON-B**	1.9	**1.3**	**93.03**	25.47	27.54	Negative	**CON-H**	0.4	**5.7**	**165.02**	*38.43*	27.54	Negative
	**CON-C**	0.4	**1.3**	31.75	4.60	18.35	Negative	**CON-H**	**>10**	**3.9**	**391.87**	**1782.26**	217.84	**High Positive (93.6%)**
	**CON-D**	**2.8**	**1.7**	15.95	2.23	18.35	Negative	**CON-I**	**>10**	**6.2**	**636.80**	**2373.93**	1400.02	**High Positive (96.2%)**
	**CON-E**	**4.9**	0.7	31.43	6.84	13.76	Negative	**CON-J**	**4.7**	**1.6**	2.30	2.79	18.35	Negative
	**CON-F**	**4.6**	**1.0**	28.55	5.02	18.35	Negative	**CON-K**	0.3	0.2	**109.78**	4.32	13.76	Negative
								**CON-L**	1.1	0.2	25.03	23.22	**40716.88**	**Positive (51.2%)**
	**Total Positive n (%[9])**	*3 (3.0)*	*4 (4.0)*	*2 (2.0)*	*0 (0.0)*	*0 (0.0)*	*0 (0.0)[10]*	**Total Positive n (%[9])**	*3 (2.0)*	*5 (3.4)*	*5 (3.4)*	*3 (2.0)*	*1 (0.7)*	*4 (57.1)[10]*

Abbreviations: CON, control; DNA, double-stranded DNA; ELISA, enzyme-linked immunosorbent assay; IgA, immunoglobulin A; IgG, immunoglobulin G; IU, International Units; NAB, neutralizing antibodies; N, nucleoprotein; Neg, Negative for all of Ro52, SSB/LA, Sm, U1RNP, Ribosomal P, and dsDNA; OD, optical density; RBD, receptor binding domain of S1 protein; S1, SRS-CoV2 spike protein (S1 subunit); SLE, systemic lupus erythematosus; xMAP®, addressable laser bead immunoassay (Luminex Corp).

[1] Cutoff for positivity: 1.9 OD Ratio

[2] Cutoff for positivity: 0.8 OD Ratio

[3] XMAP anti-N cutoff for positivity: 92.48 IU/mL, which is equivalent to 700 MFI

[4] XMAP anti-RBD cutoff for positivity: 201.84 IU/mL, which is equivalent to 700 MFI

[5] XMAP anti-S1 cutoff for positivity: 3839.31 IU/mL, which is equivalent to 700 MFI

[6] Cutoff for positivity: 20%

[7] of cohort (n = 173)

[8] Only SLE patients positive for SARS-CoV-2 antibodies pre- and/or intra-pandemic were assessed for neutralizing antibodies (pre-pandemic, n = 0; intra-pandemic, n = 6)

[9] of controls (pre-pandemic, n = 100; intra-pandemic, n = 148)

[10] Only controls positive for SARS-CoV-2 antibodies pre- and/or intra-pandemic were assessed for neutralizing antibodies (pre-pandemic, n = 6; intra-pandemic, n = 7)

Supplemental Table 3. Summary of the number of SLE patients positive for each of the SLE-related autoantibodies for those patients who developed SARS-CoV-2 serology and those who did not develop SARS-CoV-2 serology, pre- and intra-pandemic, with the corresponding Chi-squared p-values.

	**Pre-Pandemic SLE antibody assessment**			**Intra-Pandemic SLE Antibody Assessment**		
	**N positive SARS-CoV-2 Serology+ (%)**	**N Positive SARS-CoV-2 Serology- (%)**	**P Value**	**N positive SARS-CoV-2 Serology+ (%)**	**N Positive SARS-CoV-2 Serology- (%)**	**P Value**
**Anti-Ro52**	3 (60%)[1]	48 (34.8%)[3]	0.496	3 (50%)[8]	49 (30.4%)[9]	0.571
**Anti-SSA/Ro60**	0[1]	48 (33.6%)[4]	0.276	1 (16.7%)[8]	57 (35.2%)[10]	0.617
**Anti-SSB/La**	0[1]	16 (11.2%)[4]	0.953	1 (16.7%)[8]	14 (8.7%)[9]	1.000
**Anti-Sm**	0[1]	12 (8.3%)[5]	1.000	0[8]	13 (8.1%)[9]	1.000
**Anti-U1RNP**	1 (20%)[1]	36 (25.0%)[5]	1.000	2 (33.3%)[8]	44 (27.3%)[9]	1.000
**Anti-Ribosomal P**	0[1]	7 (5.1%)[6]	1.000	0[8]	14 (8.8%)[11]	0.993
**Anti-dsDNA**	1 (16.7%)[2]	52 (32.7%)[7]	0.704	1 (16.7%)[8]	48 (30.8%)[12]	0.776

Abbreviations: dsDNA, double stranded DNA; RNP, U1 ribonucleoprotein; Ro5, tripartite motif 21 antigen; Sm, Smith U2-U6 RNP antigen; SSA, Sjögren syndrome antigen A or Ro60; SSB, Sjögren syndrome antigen B or La

1- Five of the 6 patients who developed SARS-CoV-2 serology had pre-pandemic samples that were tested for anti-Ro52, anti-SSA/Ro60, anti-SSB/LA, Anti-Sm, Anti-U1RNP and anti-Ribosomal P.
2- All 6 of the patients who developed SARS-CoV-2 serology had pre-pandemic samples that were tested for anti-dsDNA.
3- 138 of the pre-pandemic SARS-CoV-2 serology negative samples were tested for anti-Ro52.
4- 143 of the pre-pandemic SARS-CoV-2 serology negative samples were tested for anti-SSA/Ro60 and anti-SSB/LA.
5- 144 of the pre-pandemic SARS-CoV-2 serology negative samples were tested for anti-Sm and anti-U1RNP.
6- 137 of the pre-pandemic SARS-CoV-2 serology negative samples were tested for anti-Ribosomal P.
7- 159 of the pre-pandemic SARS-CoV-2 serology negative samples were tested for anti-dsDNA.
8- All 6 of the patients who developed SARS-CoV-2 serology had intra-pandemic samples that were tested for anti-Ro52, anti-SSA/Ro60, anti-SSB/LA, anti-Sm, Anti-U1RNP, anti-Ribosomal P and anti-dsDNA.
9- 161 of the intra-pandemic SARS-CoV-2 serology negative samples were tested for anti-Ro52, anti-SSB/LA, anti-Sm and anti-U1RNP.
10- 162 of the intra-pandemic SARS-CoV-2 serology negative samples were tested for anti-SSA/Ro60.
11- 160 of the intra-pandemic SARS-CoV-2 serology negative samples were tested for anti-Ribosomal P.
12- 156 of the intra-pandemic SARS-CoV-2 serology negative samples were tested for anti-dsDNA.
